# Therapeutic singing-induced swallowing exercise for dysphagia in advanced-stage Parkinson’s disease

**DOI:** 10.3389/fneur.2024.1323703

**Published:** 2024-04-02

**Authors:** Myung Sun Yeo, Jihye Hwang, Hye Kyoung Lee, Soo Ji Kim, Sung-Rae Cho

**Affiliations:** ^1^Music Therapy Education, Graduate School of Education, Ewha Womans University, Seoul, Republic of Korea; ^2^Department and Research Institute of Rehabilitation Medicine, Yonsei University College of Medicine, Seoul, Republic of Korea; ^3^Graduate Program of Biomedical Engineering, Yonsei University College of Medicine, Seoul, Republic of Korea; ^4^Brain Korea 21 Plus Project for Medical Science, Yonsei University College of Medicine, Seoul, Republic of Korea; ^5^Rehabilitation Institute of Neuromuscular Disease, Yonsei University College of Medicine, Seoul, Republic of Korea

**Keywords:** therapeutic singing, swallowing exercise, dysphagia, advanced stage, Parkinson’s disease

## Abstract

**Background:**

With longer life spans and medical advancements, the rising number of patients with advanced-stage Parkinson’s disease (PD) warrants attention. Current literature predominantly addresses dementia and fall management in these patients. However, exploring the impact of swallowing function on patients with advanced PD is crucial. Previous research has demonstrated notable enhancements in the quality of life related to voice for participants following a group singing-intervention program. To further elucidate the effect of individual singing-induced swallowing exercises, our study aimed to investigate the quantitative and qualitative effects of therapeutic singing on swallowing function in patients with advanced PD in comparison to a matched usual care control group. The hypothesis of this study is that therapeutic singing-induced swallowing exercises can assist to maintain swallowing function in patients with advanced PD.

**Methods:**

This prospective matched control study compared the effects of a 6-week therapeutic singing-based swallowing intervention on swallowing function and quality of life in patients with advanced PD. The intervention group received individual sessions with a music therapist and conventional individual physical therapy. The control group received the same standard physical therapy for 6 weeks without music intervention. The primary outcome measure was Video Fluoroscopic Dysphagia Scale (VDS).

**Results:**

The study revealed that the intervention group maintained swallowing function, whereas the control group experienced deterioration, indicating significant time-dependent changes in Penetration-Aspiration Scale (PAS), National Institutes of Health-Swallowing Safety Scale (NIH-SSS), and VDS. Analysis of PAS and NIH-SSS liquid food scores in both groups showed significant time effects. However, the intervention group exhibited no significant differences between the pre- and post-tests, indicating preservation of the swallowing function. VDS of liquid food indicated an interaction effect between time and group in the pharyngeal phase and total scores. The Swallowing-Quality of Life showed significant time-effect improvement in the intervention group.

**Conclusion:**

Therapeutic singing exercises may help maintain swallowing function in advanced PD patients, potentially enhancing quality of life related to swallowing in those with advanced-stage diseases.

**Clinical trial registration:**

https://cris.nih.go.kr/cris/search/listDetail.do, identifier KCT0008644.

## 1 Introduction

Advanced-stage Parkinson’s disease (PD), defined as stages 4 and 5 on the Hoehn and Yahr scale, (1) is characterized by the deterioration of key functions including ambulatory dysfunction, worsening imbalance, and swallowing impairment (2, 3). It also includes the progression of non-motor symptoms and medication-induced adverse effects accelerating the decline in the quality of life (4). Declining efficacy of medications results from the diminishing dopaminergic neurons in the substantia nigra and a progressively lower capacity to store and convert exogenous dopamine (5). Despite variations in the predominance and severity of clinical phenotypes in advanced-stage PD, disability in the later stages is dominated by levodopa resistance and end-of-dose failure (6, 7). Current literature presents few clinical studies with patients entering the late disease stage, (8) leading to a lack of consensus because of ambiguous clinical characteristics in advanced and late-stage PD and unfavorable prognosis as the disease progresses to death (6). While improved general healthcare has increased longevity and improved clinical management of PD, the prevalence of advanced stage PD is expected to accelerate in the future. Therefore, more intensive and individualized interventions are required to address the complexity of the disease (9, 10).

Among the symptoms evident in the progression of PD, dysphagia is present in every stage of the disease; however, it becomes predominant in the advanced stages (11). PD symptoms lead to abnormal muscle movements causing oral and oropharyngeal dysfunction, (12, 13) and dysphagia often worsens with disease progression (14, 15). Thus, dysphagia emerges as a major cause of mortality and morbidity due to serious complications associated with dehydration, malnutrition and aspiration pneumonia (16).

Few therapeutic options, including levodopa optimization, are available for patients with advanced PD (17). However, dysphagia and dyskinesia are poorly controlled by existing drugs (18). Therefore, rehabilitative therapies are crucial to slow disease progression. Therapeutic strategies involving expiratory muscle strength training or electrical stimulation have shown improvements in degenerative function (coordination, speed, and volume), quality of life, and social relationships in individuals with PD (19). Previous literatures have highlighted the clinical assessment and therapeutic management of PD patients, often focusing on falls, postural instability, urinary dysfunction, freezing, bradykinesia, dysarthria, choking, dementia, psychosis, excessive daytime sleepiness, apathy, depression, and anxiety (20, 21). However, less interest has been channeled toward managing health-related quality of life in patients with advanced PD who are more likely to experience lifelong swallowing disabilities. Other studies have reported that the severity of dysphagia has a negative impact on an individual’s quality of life (22). However, interventions targeting dysphagia have shown mixed effects on quality of life, with some articles reporting improvements that are not consistent across all interventions (23). For instance, enteral tube feeding, which is beneficial for maintaining physical health, also has drawbacks, as it leads to feelings of isolation (24, 25). Similarly, texture-modified food had both positive and negative effects on quality of life, as the appearance of such foods made individuals feel self-conscious and excluded from others (26). Prioritizing the treatment of non-motor complications, including dysphagia, is thus essential.

As a non-pharmacological intervention approach, a positive impact of a singing-integrated intervention on swallowing function in individuals with PD has been reported (27–29). Singing as a musically coordinated sensorimotor activity has been identified as a promising intervention considering its involvement in breathing, vocalization, and swallowing based on anatomical and neural correlation (30, 31). Singing requires increased control of respiratory, articulatory, and vocal organs (32, 33), and the process of singing intervention in our study has been modified to promote the laryngeal and articulatory movements that are involved in swallowing (34).

Significant improvements in swallowing function have been reported in previous studies after the completion of therapeutic singing-induced swallowing exercises in individuals with PD or head and neck cancer (27–29). Considering the additional benefits of singing such as emotional support and quality of life (35). Therapeutic singing-induced swallowing exercise could provide a comprehensive approach for patients with advanced PD. The hypothesis of this study is that therapeutic singing-induced swallowing exercises can assist to maintain swallowing function in patients with advanced PD. This study therefore aimed to investigate the quantitative and qualitative effects of therapeutic singing on swallowing function in patients with advanced PD in comparison to a matched usual care control group on the primary outcome of VDS liquid food total score. We also aimed to determine the results on secondary measures of PAS, NIH-SSS, and SWAL-QOL.

## 2 Materials and methods

### 2.1 Study design and ethics

This prospective intervention study with a matched control group was approved by the Institutional Review Board of Yonsei University Health System (Approval No. 4–2012-0483, Approval No. 4–2022-0560) at Severance Hospital and was conducted in accordance with the Declaration of Helsinki. The study was registered under the Clinical Research Information Service (CRIS) with trial registration number KCT0008644. All participants provided written informed consent after the rights of the participants and the purpose, methods, benefits, and risks of the study were fully explained. The study was designed as a matched control study, in which one researcher aiming to align the control group with the intervention group based on sex, age, diagnostic presentation, and degree of severity.

### 2.2 Participants and data source

**Figure 1:**
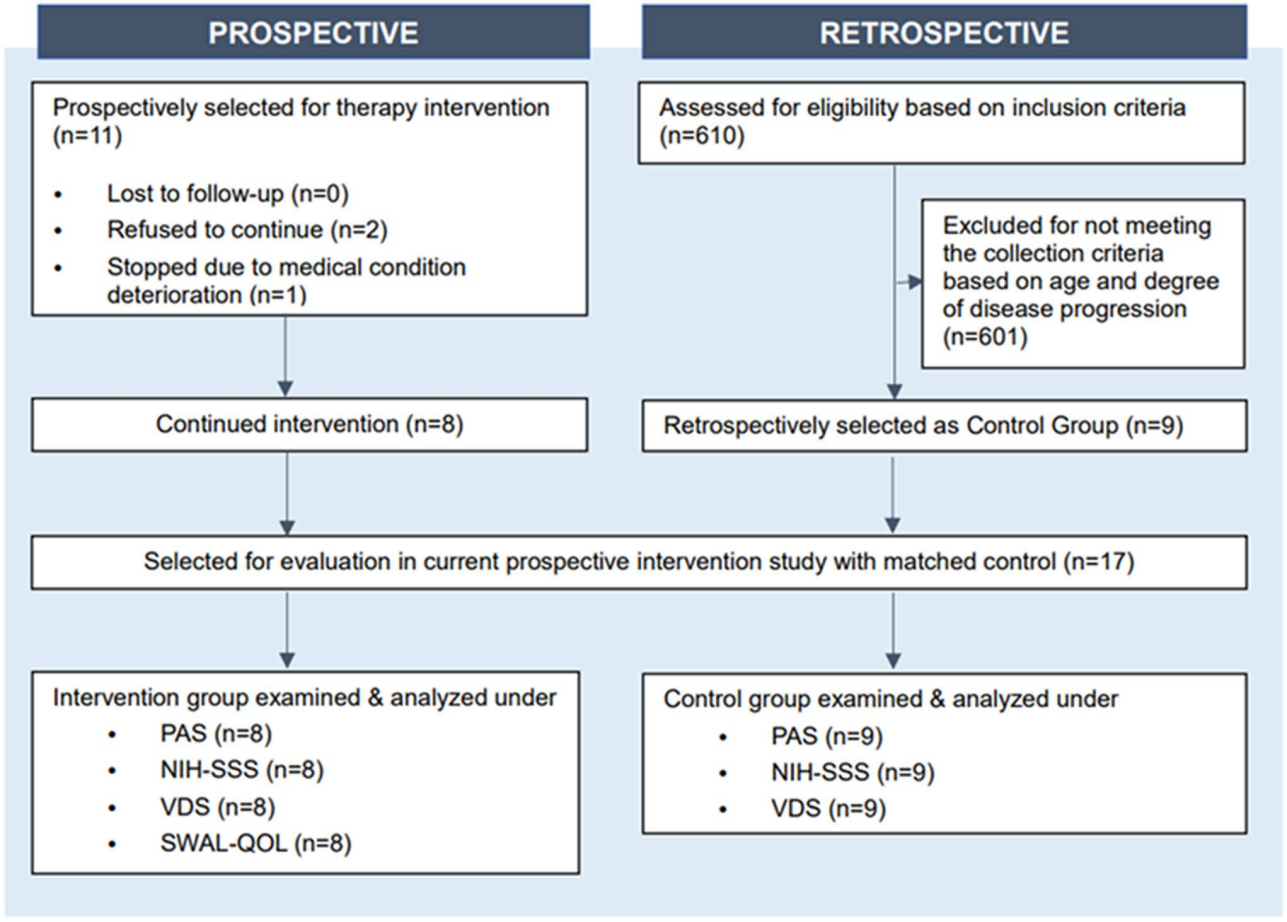
Patient disposition graph.

Initially 11 patients were selected for therapy intervention; two of them autonomously declined to continue participation and one stopped the intervention due to deterioration of their medical condition (Figure 1). Therefore, eight patients with advanced PD completed the full course of therapeutic singing-induced swallowing exercises. All participants in the intervention group were recruited from the same rehabilitation hospital, where they provided legal consent to participate. The inclusion criteria for all participants of this study were (1) diagnosis of Parkinson’s disease and Parkinsonism, (2) at or above stage 4 of the Hoehn and Yahr scale during the period of receiving PD-related medication and their usual antiparkinsonian treatment, (3) over 60 years of age, (4) no hearing impairment, and (5) no severe cognitive impairment. Matched control participants were selected from historical records. Six hundred ten patients admitted to the Severance Hospital, Seoul, South Korea, who received conventional therapy (excluding singing therapy) between 2017 and 2022 were reviewed and nine met the study inclusion criteria. Matching was performed by sex, age (±1 years), disease duration (±1 years), Hoehn and Yahr scale, and Modified Barthel Index score at initiation of admission, and swallowing function evaluation, as identified from Electronic Medical Record (Figure 1).

The target sample size was based on the results of another small music intervention study involving patients with PD (*n* = 13) with a similar dosage (36). To achieve an effect size of 0.60 with a fixed alpha error of 0.05 and power of 0.80 would require a sample size of 20.

### 2.3 Therapeutic singing-induced swallowing exercise

The intervention consisted of a 6-week program in which participants individually attended sessions with a music therapist twice a week for 30 min. This was in addition to the conventional therapy, which the control group also received. Conventional therapy was also administered twice a week for 30 min over 6 weeks. The control group followed traditional rehabilitation offers as scheduled. The components of this rehabilitation intervention included physical and occupational therapies.

The therapeutic singing-based swallowing intervention consisted of five parts as indicated in Supplementary Table 1: (1) respiratory muscle relaxation, (2) vocal warm-up, (3) vocal exercise for laryngeal elevation, and (4) modified singing. Step 1: Respiratory muscle relaxation involves stretching the neck muscles, lifting, and lowering the shoulders, and stretching the arms while participants maintain trunk balance. All movements in this step were accompanied by live electronic piano playing by a certified music therapist, and the dynamics of the music were adjusted to the participant’s movement. Step 2: Vocal warm-up involves vocalizing single vowels, holding the breath for seconds, humming, and gliding a sound. The purpose of this stage is vocal fold relaxation, breathing control, and laryngeal movement. Step 3: Vocal exercises for laryngeal elevation consisted of singing two different notes from lower to higher pitches in a sequence with chord progression. Step 4: Modified singing focused on respiration (e.g., breathing control). Certified music therapists performed all intervention processes. The baseline measurement was assessed 1 week before the first session of the therapeutic singing intervention, and the post-test was assessed within 1 week after the intervention was terminated. Due to COVID19 our design was modified, requiring participants to strictly adhere to hospital policies of wearing masks during the physical preparation time. The participants also wore a transparent plastic face shield during therapy to prevent the spread of aerosols, including saliva. More time was needed to ensure that patients did not feel uncomfortable due to this additional personal protection equipment, and also that it did not hinder the treatment procedure.

The focus of our study on dysphagia rehabilitation centered on actions that involve the opening of the jaw. This emphasis stems from the understanding that the muscles responsible for hyolaryngeal elevation, corresponding to those involved in jaw-opening, play a crucial role in enhancing laryngeal elevation, similar to the effects of traditional swallowing exercises. Additionally, in steps 2 and 3 of our therapeutic singing intervention, patients are required to hum and glide across various pitch ranges, necessitating the precise control of their laryngeal muscles (Supplementary Table 1). A related source supports this by noting that “effortful pitch glide” induces notably greater excursions in anterior hyoid, superior hyoid, hyolaryngeal approximation, laryngeal elevation, and lateral pharyngeal wall medialization compared to swallowing (37). When participants underwent the pharyngeal squeeze maneuver that involved a forceful sound of “ee,” the contraction of the pharyngeal constrictors allows improvement of pharyngeal strength (37). These findings suggest that the effortful pitch glide could be an effective exercise targeting crucial swallowing muscles, particularly the long pharyngeal muscles responsible for elevating the larynx and shortening the pharynx during swallowing (37). Similarly, the maneuver of pharyngeal muscles involved in singing therapy allows for these muscles to be trained and thus gain strength (37). Consequently, the mechanism of new exercise introduced in the aforementioned study supports our study design as training the muscles involved in singing can also have similar effect to swallowing exercise as they belong to the same group of muscles.

### 2.4 Outcome measures

The primary outcome measure of this study was VDS liquid food total score and the secondary outcome measures were PAS, NIH-SSS, and SWAL-QOL. Primary and secondary outcome measures were conducted at pre and post time point of 6-week intervention of therapeutic singing-induced swallowing exercise. A Video Fluoroscopy Swallowing Study was used to evaluate swallowing function in participants with PD. They performed swallowing tasks while ingesting three types of barium (one liquid and two solid powders). Participants in all groups swallowed 5 mL of three types of solution mixed with barium sulfate according to the instructions of the tester at the start of evaluation (12% semisolid solution consisted of barium sulfate, yogurt powder 9 g, Baritop HD power 9 g and 150 mL of water. 6% semisolid mixture consisted of barium sulfate, yogurt powder 4.5 g, Baritop HD power 4.5 g and 150 mL of water) (38). The outcomes of the videofluoroscopy swallowing study were evaluated using the penetration-aspiration scale (PAS), National Institutes of Health-Swallowing Safety Scale (NIH-SSS), and the video fluoroscopic dysphagia scale (VDS).

The PAS quantifies the degree of penetration and aspiration observed during the Video Fluoroscopy Swallowing Study and consists of an eight-point scale that assesses the depth of bolus passage into the airway and the patient’s response. A higher PAS score indicates more severe symptoms (39). The PAS was used to evaluate changes in swallowing function. The NIH-SSS assesses swallowing stability by evaluating food residue, laryngeal penetration, aspiration response, maximal esophageal entry, and multiple swallows based on videofluoroscopic swallowing study observations (40). A higher score indicate more severe dysphagia (40). The NIH-SSS was used solely in the intervention group to assess changes in the swallowing function.

The VDS is a 14-item scale that assesses the oropharyngeal function during swallowing (41). The items included lip closure, bolus formation, mastication, apraxia, tongue-to-palate contact, premature bolus loss, oral transit time, pharyngeal swallowing triggering, vallecular residue, laryngeal elevation, pyriform sinus residue, coating of the pharyngeal wall, pharyngeal transit time, and aspiration (41). The first seven items assess the oral phase, whereas the next seven items evaluate the pharyngeal phase (41). The VDS scores range from 0 to 100, with higher scores indicating poorer swallowing function (41).

The SWAL-QOL was used to assess the quality of life of patients with oropharyngeal swallowing disorders (42). It consists of 11 subcategories and 44 questions (42). Higher scores indicate a higher quality of life associated with swallowing impairment (42). Two separate licensed physiatrists and two separate researchers with expertise in the related field conducted the outcome measures, and inter-rater reliability was tested.

### 2.5 Statistical analysis

Participants with advanced PD who underwent therapeutic singing-induced swallowing exercises were retrospectively compared with matched controls. Two-way repeated measures ANOVA and paired t-tests were used to analyze changes in swallowing functions over 6 weeks for each group and compare differences (*p* < 0.05 was set as criterion for statistical significance).

## 3 Results

### 3.1 Participants

After evaluating their eligibility for the study, the participating patients were divided into the intervention (*N* = 8) and control (*N* = 9) groups (Figure 1). The mean age of the participants in the intervention was 71.88 years (range of 56–86) with a mean onset duration of motor symptoms of 8.86 years and onset duration of dysphagia of 2.76 years. The mean age of the participants in the control group was 65.44 years (range–53–86) with a mean onset duration of 3.00 years. The level of disability of all participants was assessed using the Hoehn and Yahr scale. There were no significant differences between the intervention and control groups in terms of sex (*p* = 0.229), age (*p* = 0.277), duration of motor symptom onset (*p* = 0.923), duration of dysphagia onset (*p* = 0.960), Hoehn and Yahr stage (*p* = 0.819), or Modified Barthel Index total score (*p* = 0.847) (Table 1). The Modified Barthel Index score criteria also did not show any statistical difference between the two groups (Table 1).

**Table 1:** Clinical characteristics of participants.

Characteristics	Intervention group (*n* = 8)	Control group (*n* = 9)	*p*-value
Gender			
Male, *N* (%)	5 (62.50%)	3 (33.33%)	0.229
Female, *N* (%)	3 (37.50%)	6 (66.67%)	0.229
Age (years), (mean ± SEM)	71.88 ± 3.54	65.44 ± 4.09	0.277
Onset duration, years			
Motor Symptom	8.86 ± 2.04	8.89 ± 1.76	0.923
Dysphagia	2.76 ± 1.42	1.78 ± 0.55	0.960
Hoehn and Yahr stage, *N* (%)			
Stage 4	4 (50.00%)	4 (44.44%)	0.819
Stage 5	4 (50.00%)	5 (55.56%)	0.819
Modified Barthel Index			
Personal hygiene	3.38 ± 0.56	2.78 ± 0.68	0.619
Bathing self	1.75 ± 0.49	2.22 ± 0.68	0.619
Feeding	6.13 ± 1.09	5.00 ± 1.52	0.767
Toilet	4.38 ± 1.02	4.56 ± 1.37	0.921
Stair climbing	3.13 ± 1.08	4.78 ± 1.45	0.398
Dressing	6.13 ± 0.79	6.00 ± 1.19	0.960
Bowel control	7.63 ± 1.25	5.67 ± 1.51	0.363
Bladder control	6.63 ± 1.25	5.56 ± 1.63	0.763
Ambulation	7.25 ± 1.68	7.89 ± 1.93	0.729
Chair/bed transfer	9.38 ± 1.61	9.33 ± 2.07	0.802
Total score	55.38 ± 9.74	53.78 ± 13.32	0.847

Data are presented as mean ± SEM or *n* (%). MBI, Modified Barthel Index, score range of 0 to 100. FIM, Functional Independence Measure, score range of 18 to 126. Lower scores of Modified Barthel Index and Functional Independence Measure indicate higher degree of dependence on caregivers while carrying out activities of daily living.

### 3.2 Swallowing function

The PAS, NIH-SSS, and VDS scores were obtained during the videofluoroscopy swallowing study. Two independent scorers had assessed PAS, NIH-SSS, and VDS for the nine patients in the control group (during their follow-up earlier), and the same scorers evaluated the scores for the eight participants in the intervention group later. The inter-rater reliabilities of the PAS, NIH-SSS, and VDS demonstrated intra-class correlation coefficients (ICC) of 1.00, 0.90, and 0.99, respectively. When comparing changes in the PAS and NIH-SSS scores between the intervention and control groups, both scales exhibited a significant time-effect before and after treatment for liquid food scores (*F*_1,_ = 6.480, *p* = 0.022; *F*_1,_ = 4.745, *p* = 0.046) (Table 2). The results indicated that the swallowing function of the control group weakened, showing significant time-dependent changes in the PAS and NIH-SSS in the pre- and post-treatment assessments (*p* = 0.045, *p* = 0.009, respectively, by Paired *T*-test). However, the results showed no significant differences between the pre-test and post-test PAS and NIH-SSS liquid substance scores in the intervention group, indicating that the swallowing function of the intervention group was maintained (*p* = 0.173, *p* = 0.732 by Paired *T*-test) (Table 2).

**Table 2:** Swallowing functions in Parkinson’s disease patients with dysphagia after singing-induced swallowing exercises.

Variables	Intervention group		Control group		Time		Group		Time * Group	
	Pre	Post	Pre	Post	F	*p*	F	*p*	F	*p*
PAS										
12% semisolid food	1.88 ± 0.88	1.00 ± 0.00	2.33 ± 0.88	1.89 ± 0.65	0.870	0.366	0.871	0.366	0.093	0.765
6% semisolid food	2.00 ± 0.87	1.50 ± 0.33	2.44 ± 0.87	2.00 ± 0.65	0.743	0.402	0.301	0.591	0.003	0.960
Liquid food	2.63 ± 0.65	3.63 ± 1.02	3.56 ± 0.96	5.67 ± 0.88^#^	6.480	0.022^*^	1.793	0.201	0.827	0.378
NIH-SSS										
12% semisolid food	3.25 ± 0.31	3.13 ± 0.13	3.78 ± 0.43	3.78 ± 0.36	0.144	0.709	1.700	0.212	0.144	0.709
6% semisolid food	3.88 ± 0.30	3.75 ± 0.25	3.89 ± 0.39	4.00 ± 0.44	0.002	0.964	0.073	0.791	0.614	0.445
Liquid food	4.13 ± 0.35	4.25 ± 0.25	4.11 ± 0.39	5.00 ± 0.33^##^	4.745	0.046^*^	0.769	0.394	2.693	0.122

Note. Data are presented as mean ± SEM. PAS, Penetration-Aspiration Scale with score range of 1 to 8; NIH-SSS, NIH-Swallowing Safety Scale with score range of 0 to 42. Higher scores indicate worsening of symptoms for PAS and NIH-SSS. ^*^p < 0.05 significant effect by two-way repeated-measures ANOVA. ^#^*p* < 0.05, ^##^*p* < 0.01 significant difference by Paired *T*-test. Data are presented as mean ± SEM.

The VDS of liquid food showed an interaction effect between time and group in the pharyngeal phase and the total scores (*F* = 9.425, *p* = 0.008; *F* = 5.859, *p* = 0.029) (Table 3). The VDS of liquid food also showed time effect in the pharyngeal phase and the total scores (*F* = 15.017, *p* = 0.001; *F* = 14.864, *p* = 0.002) (Table 3). The results showed significant decrease in function over time in the pharyngeal phase and total VDS scores of the control group (*p* < 0.001), indicating impaired swallowing function in the control group, especially in the pharyngeal phase (pharyngeal phase: *p* = 0.642; total score: *p* = 0.439 by Paired *T*-test). Conversely, the swallowing function of the intervention group was maintained, with no significant differences between the pre- and post-test pharyngeal phases and total VDS scores (Table 3). Each VDS parameter was also analyzed to identify specific phase parameters influenced by the intervention, which revealed no significant change in the oral phase. In the pharyngeal phase, the VDS scores of coating on the pharyngeal wall showed a time-effect (*F* = 5.674, *p* = 0.031), interaction effect (*F* = 5.674, *p* = 0.031), with no group effect (*F* = 1.471, *p* = 0.244), and the results indicated that there was a significant difference between pre- and post-test scores of coating on the pharyngeal wall in the control group (*p* = 0.035 by Paired *T*-test), whereas there was no difference in the intervention group (*p* = 0.351 by Paired *T*-test) (Table 4). Pharyngeal transit time showed a group effect (*F* = 4.706, *p* = 0.047), but did not show time effect (*F* = 2.017, *p* = 0.176) and interaction effect (*F* = 2.017, *p* = 0.176) (Table 4). However, this should be carefully interpreted because of the lack of statistical significance of the time-dependent changes in the intervention group. Prior to the commencement of treatment, there was no significant difference in the pharyngeal transit time scores between the control group and the intervention group (*p* = 0.169 by independent *T*-test). However, following 6 weeks of music therapy, a noteworthy delay in pharyngeal transit time was observed in the control group compared to the intervention group (*p* = 0.031 by independent *T*-test). The aspiration results indicated that the swallowing function in the control group was significantly weakened (*p* = 0.022 by Paired *T*-test), especially in the pharyngeal phase, whereas the swallowing function in the intervention group was maintained (Tables 3, 4).

**Table 3:** Swallowing functions in Parkinson’s disease patients with dysphagia after singing-induced swallowing exercises.

VDS	Intervention group		Control group		Time		Group	Time * Group		
	Pre	Post	Pre	Post	*F*	*p*	*F*	*p*	*F*	*p*
12% semisolid food										
Oral phase	11.13 ± 4.01	10.56 ± 3.60	14.00 ± 4.09	16.39 ± 4.42	0.813	0.381	0.586	0.456	2.123	0.166
Pharyngeal phase	20.44 ± 4.41	26.69 ± 4.11	20.72 ± 5.24	22.78 ± 4.00	3.685	0.074	0.091	0.767	0.940	0.348
Total score	31.56 ± 7.51	37.25 ± 6.98	34.72 ± 8.13	39.17 ± 7.89	4.039	0.063	0.057	0.814	0.061	0.809
6% semisolid food										
Oral phase	11.13 ± 4.01	12.00 ± 3.97	13.67 ± 4.21	15.28 ± 4.66	1.251	0.281	0.241	0.631	0.110	0.745
Pharyngeal phase	24.25 ± 4.72	26.88 ± 3.97	22.50 ± 4.87	28.11 ± 4.15^#^	9.077	0.009^**^	0.002	0.967	1.193	0.292
Total score	35.38 ± 7.64	38.88 ± 6.82	36.17 ± 7.72	43.39 ± 7.99^#^	7.999	0.013^*^	0.062	0.806	0.964	0.342
Liquid food										
Oral phase	7.88 ± 3.67	10.13 ± 3.99	13.00 ± 3.97	15.72 ± 4.54	3.644	0.076	0.903	0.357	0.033	0.859
Pharyngeal phase	27.31 ± 3.38	28.88 ± 4.41	22.67 ± 5.34	36.44 ± 4.22^###^	14.864	0.002^**^	0.059	0.811	9.425	0.008^*^
Total score	35.19 ± 5.90	39.00 ± 6.70	35.67 ± 7.55	52.17 ± 7.31^###^	15.017	0.001^**^	0.513	0.485	5.859	0.029^*^

Data are presented as mean ± SEM. VDS, Videofluoroscopic Dysphagia Scale, with total score range of 0 to 100. Higher scores indicate worsening of symptoms. ^*^p < 0.05, ^**^p < 0.01 significant effect by two-way repeated measures ANOVA. ^#^*p* < 0.05, ^###^*p* < 0.001 significant difference by Paired *T*-test.

**Table 4:** Swallowing functions in Parkinson’s disease patients with dysphagia after singing-induced swallowing exercises.

VDS	Intervention group		Control group		Time		Group		Time * Group	
	Pre	Post	Pre	Post	*F*	*p*	*F*	*p*	*F*	*p*
Lip closure	0.25 ± 0.25	0.50 ± 0.33	0.00 ± 0.00	0.00 ± 0.00	1.134	0.304	2.306	0.150	1.134	0.304
Bolus formation	2.25 ± 0.94	2.63 ± 1.05	2.67 ± 0.93	3.00 ± 0.87	0.941	0.347	0.095	0.763	0.003	0.955
Mastication	2.50 ± 1.30	3.00 ± 1.46	3.56 ± 1.24	4.00 ± 1.15	0.941	0.347	0.344	0.566	0.003	0.955
Apraxia	0.00 ± 0.00	0.19 ± 0.19	0.17 ± 0.17	0.33 ± 0.33	2.008	0.177	0.311	0.585	0.007	0.935
Tongues to palate contact	0.63 ± 0.63	0.63 ± 0.63	2.78 ± 0.88	3.89 ± 1.39	2.017	0.176	4.261	0.057	2.017	0.176
Premature bolus loss	1.88 ± 0.74	2.44 ± 0.75	2.50 ± 0.79	2.83 ± 0.73	1.982	0.180	0.251	0.623	0.130	0.724
Oral transit time	0.38 ± 0.38	0.75 ± 0.49	1.33 ± 0.53	1.67 ± 0.53	2.008	0.177	2.090	0.169	0.007	0.935
Triggering of pharyngeal swallow	0.56 ± 0.56	0.56 ± 0.56	2.00 ± 0.79	2.50 ± 0.79	0.882	0.362	3.107	0.098	0.882	0.362
Vallecular residue	2.00 ± 0.00	2.25 ± 0.25	2.67 ± 0.33	3.11 ± 0.48	3.151	0.096	3.151	0.096	0.247	0.626
Laryngeal elevation	5.63 ± 1.65	5.63 ± 1.65	2.00 ± 1.32	5.00 ± 1.58	1.765	0.204	1.281	0.275	1.765	0.204
Pyriform sinus residue	4.50 ± 0.00	5.06 ± 0.56	6.00 ± 0.75	6.50 ± 0.79	2.008	0.177	3.211	0.093	0.007	0.935
Coating on the pharyngeal wall	7.88 ± 1.13	7.88 ± 1.13	4.00 ± 1.58	8.00 ± 1.00^#^	5.647	0.031^*^	1.471	0.244	5.647	0.031^*^
Pharyngeal transit time	0.00 ± 0.00	0.00 ± 0.00	1.33 ± 0.88	2.67 ± 1.05	2.017	0.176	4.706	0.047^*^	2.017	0.176
Aspiration	6.75 ± 1.36	7.50 ± 1.88	4.67 ± 1.67	8.67 ± 1.45^#^	4.486	0.051	0.054	0.819	2.100	0.168

Data are presented as mean ± SEM. VDS, Video fluoroscopic Dysphagia Scale, with total score range of 0 to 100. Higher scores indicate worsening of symptoms. ^*^p < 0.05 significant effect by two-way repeated-measures ANOVA. ^#^*p* < 0.05 significant difference by Paired *T*-test.

SWAL-QOL was measured only in the intervention group. The largest observed difference was found in the symptom frequency between pre- and post-intervention, although this did not reach statistical significance. Additionally, a significant difference between the pre-test and post-test was observed for the total SWAL-QOL score (*p* = 0.005), including subcategories such as food selection (*p* = 0.015), fear (*p* = 0.020), mental health (*p* = 0.009), social functioning (*p* = 0.036), and fatigue (*p* = 0.015), as shown in Table 5.

**Table 5:** SWAL-QOL scores before and after singing-induced swallowing exercises for intervention group (*N* = 8).

Parameter	Pre	Post	*t*	*p*
Burden	7.25 ± 1.48	7.38 ± 0.74	−0.196	0.850
Eating duration	6.13 ± 1.55	7.13 ± 1.55	−1.080	0.316
Eating desire	9.63 ± 2.06	9.63 ± 2.20	0.000	1.000
Symptom frequency	40.25 ± 5.44	49.88 ± 1 4.63	−0.2.064	0.078
Food selection	4.63 ± 1.76	6.63 ± 0.91	−3.191	0.015^*^
Communication	5.00 ± 2.50	6.00 ± 1.41	−1.366	0.214
Fear	9.13 ± 3.04	12.88 ± 2.10	−0.794	0.020^*^
Mental health	10.63 ± 2.72	15.25 ± 2.18	−0.3.572	0.009^**^
Social functioning	12.13 ± 2.69	15.63 ± 2.44	−2.593	0.036^*^
Fatigue	5.75 ± 2.37	8.00 ± 1.06	−3.211	0.015^*^
Sleep	4.13 ± 1.55	5.13 ± 1.80	−1.038	0.334
Total	114.63 ± 12.10	143.50 ± 17.92	−3.998	0.005^**^

Data are presented as mean ± SEM. SWAL-QOL, Swallowing Quality of life questionnaire, with total score range of 0 to 100. Lower scores indicate higher degree of impairment in the quality of life. ^*^p < 0.05, ^**^p < 0.01 significant effect by two-way repeated measures ANOVA.

## 4 Discussion

The present study examined the effects of therapeutic singing-induced swallowing exercises on the PAS, NIH-SSS, VDS, and SWAL-QOL in patients with advanced PD. This is the first study to explore the effects of singing-integrated intervention on swallowing function in patients with advanced PD using a prospective intervention study with matched control group. The findings of this study indicated that minimal changes were observed in most parameters between the pre-test and post-test assessments in both the intervention and control groups. However, a clinically significant observation was made regarding the scores in the liquid food condition, serving as an indicator of a higher risk of aspiration. The intervention group exhibited stable scores in this condition, whereas the control group demonstrated a statistically significant decline in functional ability in both the PAS and NIH-SSS under liquid condition testing.

VDS is a comprehensive tool utilized in Video Fluoroscopic Swallowing Studies (VFSS) to assess swallowing function. It evaluates oral and pharyngeal phases, penetration/aspiration, residue, and abnormalities, providing a detailed understanding of dysphagia nature and severity. Compared to PAS and NIH-SS, VDS identifies swallowing issues in advanced PD patients due to its extensive parameters. It offers objective measurements, reducing subjectivity and enabling reliable comparisons. Standardized scoring ensures consistency across clinicians and settings, fostering communication and interpretation. Widely used in clinical and research settings, VDS predicts clinical outcomes like aspiration pneumonia risk and treatment response, aiding patient management decisions. It monitors swallowing changes over time for tailored interventions and improved outcomes.

When statistical analyses are conducted to compare different dysphagia scales, the significance of the results can vary based on several factors, including the sensitivity of the scales and the number of parameters they assess. Generally, scales that evaluate a broader range of parameters related to swallowing function may yield more significant results in statistical analyses. In this case, VDS scores are more sensitive that NIH-SS, and thus have shown more significant changes between the comparing groups. Scales that assess multiple parameters of swallowing function, such as VDS, are inherently more sensitive to variations in swallowing function. This increased sensitivity allows for the detection of subtle differences or abnormalities that may not be captured by scales focusing on a narrower range of parameters, such as PAS.

Given the non-linear trajectory of symptom progression observed in patients with advanced PD (43), preservation of functional levels becomes clinically important. This significance stems from the recognition that advanced PD involves a multitude of pathological alterations within the neuromuscular structures associated with swallowing, leading to impairments across all stages of the swallowing process. Moreover, aging is associated with reduced muscle tone in the elderly population, resulting in reduced chewing and swallowing capabilities, rendering patients with PD more susceptible to dysphagia (44). Considering the severity of PD as indicated by Hoehn and Yahr stages 4 and 5 in the participants, singing interventions can be beneficial in delaying symptomatic regression.

The improvement in SWAL-QOL after 6 weeks of therapeutic singing-induced swallowing exercise showed that music experience positively influenced the participants’ quality of life. Due to the retrospective nature of the study, the analysis of SWAL-QOL scores was only feasible within the intervention group. Upon examining the subcategories of the SWAL-QOL, improvements in psychosocial indicators were observed. The positive outcomes of music therapy in various functional domains of PD have been consistently reported in previous studies (45–47). Singing has been widely acknowledged for its effectiveness in enhancing emotional aspects and communication abilities, and the SWAL-QOL findings in this study support the results of previous studies (33). A study by Tamplin et al. (33) suggests that singing by nature allows patients to express and internalize their emotions better, especially when they lack the ability to communicate their feelings actively to caregivers and healthcare providers. This renowned study by Tamplin et al. (33) has proved the significant improvements in voice-related quality of life for Parkin Song participants in terms of acoustic measures of vocal loudness, with a perceived decrease in severity of voice problem, and less negative feelings about their voice problem in addition to mood enhancement and stress relief. Singing may lead to improved mood with its physiological and neurochemical effects. Singing demonstrated a positive association with diminished cortisol, beta-endorphin, and oxytocin levels, suggesting a potential modulatory impact on both mood states and components of the immune system in this preliminary investigation involving cancer patients (48). In this study, enhancements in mood were notably pronounced among individuals with lower mental wellbeing, revealing substantial positive associations between mood changes and baseline anxiety and depression levels, alongside noteworthy negative correlations with baseline levels of wellbeing and social resilience (48). The experience of music, an intangible stimulus, has the potential to elicit sensations of joy and a desire for more, akin to the gratification associated with concrete rewards linked to the dopaminergic system in the striatum (49). This implies that singing allows individuals to express and convey their emotions through music and lyrics. Singing-induced swallowing exercise adopted in our study thus can provide a healthy outlet for emotional expression and can be particularly therapeutic for patients with advanced PD dealing with stress, depression, or other challenging emotions. In addition, engaging in singing can serve as a distraction from negative thoughts and worries. Focusing on the act of singing and the enjoyment of music can redirect attention away from stressors that these patients with advanced PD may be more sensitive towards.

A similar study by Baker et al. (50) elucidated that engaging in recreational choir singing emerges as a therapeutically relevant intervention with clinical implications for alleviating depressive symptoms in individuals with dementia within the care home setting in Australia. Although this study was designed with singing in a group, the effect of singing itself was proven to alleviate depressive mood among patients with dementia which also involve cognitive decline like advanced PD. Successful participation in singing, whether individually like in our study design or in a group, can boost self-esteem and confidence. This suggests that accomplishing musical goals and receiving positive feedback from the therapists contribute to a sense of achievement for the patients with advanced PD and similar neurodegenerative diseases.

Another study by Tamplin et al. (51) targeting people with spinal cord injury patients reported that sensations of enjoyment were notably elevated following sessions of music therapy, reinforcing earlier discoveries indicating that group singing fosters a sense of happiness, positive mood, joy, elation, and a heightened feeling. The results from their study supports the results of our study including parameters such as “fear,” “mental health” and “social functioning” in SWAL-QOL showing significant improvement. It can be inferred that participating in an intervention involving music has facilitated the experience of psychosocial support, fostering emotional bonds and promoting mental health status in participants. The greatest improvement was observed in decrease of symptom frequency in the intervention group, which includes severity of drooling and presence of excessive saliva or thick phlegm (52), although this was not statistically significant. Similar results have been reported in previous studies that used expiratory muscle strength training or speech-language therapy (53, 54).

Swallowing is a complex neural representation, involving multiple regions such as the caudal sensorimotor and lateral premotor cortex, insula, temporopolar cortex, amygdala, brainstem, and cerebellum (55). Neural activation for singing commonly occurs in the inferior precentral and postcentral gyrus, superior temporal gyrus and superior temporal gyrus, and superior temporal sulcus bilaterally, as these activated areas indicated a large shared network for motor preparation associated with vocalization, execution, and sensory feedback/control for vocal production. (33, 56). These neural processes have far-reaching implications, affecting various body systems involved in the act of swallowing. Thus, it might be interpreted that functional improvement in swallowing can promote patients’ cognitive ability (53, 54). Patients with advanced PD can experience cognitive-linguistic impairments, poor self-perception of speech and neuropsychiatric symptoms including depression and anxiety (33). This is relatable to our study as it stems from the fundamental idea of singing requiring coordination of various cognitive processes such as memory, attention, and language. Engaging in these cognitive activities can have positive effects on overall cognitive function and mental well-being of patients with advanced PD.

The act of singing also requires patients to sustain their phonation, articulation and improve their respiratory support, as elucidated by previous studies of Tamplin et al. (33). This particular study by Tamplin et al. (33) emphasized on the improvement in speech functions related to articulation and breathing control, which also shares a similar focus to our study in mitigating and potentially reversing the neurodegenerative nature of the disease as it is crucial to maintain the use of swallowing muscles that are also used in singing. This can be achieved through consistent engagement in specific vocal, respiratory, and swallowing exercises tailored for individuals with advanced PD. Singing involves controlled breathing, which can have a calming effect on the nervous system. Deep, controlled breaths can reduce physiological responses to stress and promote relaxation, and training of muscles related to speech and swallowing. Singing, therefore, can serve as a promising therapeutic modality to improve speech related functions including swallowing as it enhances related muscles.

The mean levodopa dosage prescribed between the intervention group and control group in the current study did not demonstrate a statistically significant difference, suggesting that L-dopa had no known effect on the swallow function in the current study as a covariate. The applicability and efficacy of non-pharmacological treatments for speech impairment should be considered in the management of speech disorders in patients with PD. Moreover, a higher L-dopa dose does not always improve swallowing, and non-pharmacological interventions must be prioritized (57).

The advantage of the protocol used in this study is that it emphasizes the detailed mechanisms required for singing, such as breathing, vocalization, tongue base contraction, and articulation, while enabling the integrated control of complex functions through a single singing context. Patients can repeatedly perform this integrated training process as singing training is frequently used as a form of exercise training for patients who need to improve laryngeal function, and its effectiveness has been reported in several cases (38, 58). This study has important implications for the application of the effects of laryngeal muscle movements induced by therapeutic singing to assist in maintaining swallowing function.

Motor- and non-motor symptoms unresponsive to levodopa are the most reliable predictors of nursing home placement and mortality (59, 60). Clinicians have a strong tendency to focus their attention on conservative treatment of postural instability, falls, dementia and hallucinations in patients with advanced PD, the strongest independent predictors of institutionalization and death (59, 60). The clinical significance of this study arises from the novelty of assessing and treating dysphagia and the overall quality of life related to swallowing in patients with advanced PD, where the current literature mainly focuses on motor- and non-motor symptoms, excluding dysphagia and SWAL-QOL. The nature of patients with advanced PD and the heterogeneity in their symptoms also limits clinicians from conducting reliable clinical research; however, this study has presented treatment effects on patients with verified and holistic clinical characteristics.

This study serves as an important step in elucidating the impact of therapeutic singing-induced swallowing exercises on assistance of maintaining swallowing function in patients with advanced-stage PD at an individual level. The singing-induced swallowing protocol could be a potential treatment option for dysphagia in patients with PD. The clinical importance lies in providing an intervention that allows patients with advanced PD to participate and observe positive changes. One limitation of this study is that the SWAL-QOL was only assessed in the intervention group. For a more comprehensive assessment of swallowing function and quality of life in patients with advanced PD, the SWAL-QOL should be assessed in both the control and intervention groups. Other limitations include small sample and the absence of an active control group. In a future study, more patients need to be recruited as a randomized controlled trial.

Current medical practitioners may face difficulties in discerning significant changes in swallowing symptoms among patients with advanced-stage PD. As a result, we conducted the study using a 1:1 matching design and confirmed the effect of the intervention protocol through comparison with a control group. These experiences will provide researchers with suggestions for the importance of and insights into various interventions and measures. Further research should be conducted to investigate the long-term effects of therapy and to examine the effects of therapy on the quality of life of patients with advanced PD. This may involve multiple sessions of singing intervention over a longer period, providing better insight for clinicians to determine the ideal number of sessions and the degree of singing intervention depending on the progression of the disease and the type of neurodegenerative disease.

## Data availability statement

The raw data supporting the conclusions of this article will be made available by the authors, without undue reservation.

## Ethics statement

The studies involving humans were approved by Institutional Review Board of Yonsei University Health System and registered under Clinical Research Information Service. The studies were conducted in accordance with the local legislation and institutional requirements. The participants provided their written informed consent to participate in this study.

## Author contributions

MY: Conceptualization, Data curation, Methodology, Validation, Writing – original draft, Writing – review & editing. JH: Conceptualization, Investigation, Project administration, Validation, Visualization, Writing – original draft, Writing – review & editing. HL: Conceptualization, Data curation, Formal analysis, Investigation, Validation, Visualization, Writing – review & editing. SK: Conceptualization, Data curation, Investigation, Supervision, Writing – review & editing. S-RC: Conceptualization, Investigation, Project administration, Supervision, Validation, Visualization, Writing – review & editing.
