# Supplementary material for: Therapeutic singing-induced swallowing exercise for dysphagia in advanced-stage Parkinson’s disease
Source: Front Neurol. 2024 Apr 2;15:1323703. doi: 10.3389/fneur.2024.1323703 (PMC11018993; doi:10.3389/fneur.2024.1323703)
Supplement: Supplementary file 1 [file Table_1.docx]

**Supplementary**

Supplementary Table 1. Procedure of singing-enhanced swallowing therapy

| **Step** | **Procedure** (30 min) | **Description** |
| --- | --- | --- |
| 1 | Respiratory and  muscle relaxation  (10 min) | Breathing for relaxation of respiratory  Stretching arms, turning neck, and lifting and lowering the shoulders |
| 2 | Vocal warm-up  (5 min) | Vocalizing single vowels and holding breath for seconds  Humming and gliding a sound  ** Long pharyngeal muscles responsible for elevating the larynx and shortening the pharynx during swallowing* |
| 3 | Singing exercise for  laryngeal elevation  (5 min) | Singing two-interval notes from lower to higher pitches in a sequence with chord progression: Lower pitch with /u/ and higher pitch with /i/ sound  **Engage the contraction of the pharyngeal constrictors by producing a forceful sound* |
| 4 | Modified singing  (10 min) | Singing a modified song (by music therapist): focused on respiration (breathing control) |
